# Supplementary material for: New approaches on composite designs for Response Surface Methodology
Source: PLoS One. 2024 Apr 2;19(4):e0301049. doi: 10.1371/journal.pone.0301049 (PMC10986981; doi:10.1371/journal.pone.0301049)
Supplement: Attachment — Submitted filename: Responce to referees.pdf [file pone.0301049.s001.pdf]

# Response to Reviewers

The authors would like to thank the two anonymous referees and the associate editor for their valuable comments and suggestions that improved the results and the appearance of the paper.

## Response to the editor:

*When submitting your revision, we need you to address these additional requirements.*
*1. Please ensure that your manuscript meets PLOS ONE's style requirements, including those for file naming. The PLOS ONE style templates can be found at*
*https://journals.plos.org/plosone/s/file?id=wjVg/PLOSOne_formatting_sample_main_body.pdf and*
*https://journals.plos.org/plosone/s/file?id=ba62/PLOSOne_formatting_sample_title_authors_affiliations.pdf*
*2. Please update your submission to use the PLOS LaTeX template. The template and more information on our requirements for LaTeX submissions can be found at http://journals.plos.org/plosone/s/latex.*
*3. Please amend either the abstract on the online submission form (via Edit Submission) or the abstract in the manuscript so that they are identical.*
*Additional Editor Comments:*
*This paper needs a major revision to improve the presentation.*

We thank the associate editor for all his comments and suggestions. Please note that the paper style is as the journal requested and the presentation is improved as instructed. All the referees' comments have been addressed and a detailed point to point response is provided below:

## Response to reviewer 1.

1. *I have carefully reviewed the above paper titled "New Approaches on Composite Designs for Response Surface Methodology." The authors introduce two novel composite techniques aiming to enhance design efficiency within Response Surface Methodology (RSM), with a particular emphasis on the importance of Definitive Screening Designs (DSDs) as an alternative to traditional axial points in composite designs. The construction of new design matrices involves the use of orthogonal matrices and the integration of axial components from simple orthogonal designs or block orthogonal designs, as outlined by Alrweili in 2020. Significantly, the proposed designs exhibit superior performance in terms of efficiency and robustness compared to existing designs. The paper navigates the challenges associated with creating composite designs, especially when dealing with a larger number of factors and runs.*

   We thank the referee for the favourable comment, and we agree with this view.

2. *The implementation of second-order designs, while maintaining orthogonality among main effects, introduces four distinct methods. The application of the first two methods from existing literature leads to the discovery of new designs with higher D-values, while Methods 3 and 4 introduce entirely new techniques, consistently yielding higher D-values with a simultaneous reduction in the number of runs. Detailed insights into all the designs are presented in tables, facilitating a thorough comparison with existing composite designs from the literature. Notably, the newly proposed designs are highlighted in the last row of the comparison table, showcasing higher D-values or a reduced number of runs. This suggests the potential for cost reduction in experiments while achieving desired outcomes within an acceptable time frame*

   The referee is correct. We were aiming at cost reduction in the designs and we appreciate the referees verification on this.

3. *The paper not only makes a significant contribution to the field of experimental design within Response Surface Methodology but also offers practical benefits to readers and practitioners. The innovative approaches, coupled with a comprehensive comparative analysis, provide valuable insights for researchers and professionals engaged in experimental design. The potential for cost reduction highlighted in the findings could be particularly advantageous to practitioners in various fields.*

   Yes. We agree with the referee as we also aim in helping practitioners decide which the most appropriate design to use in their experiments and that is why the tables were presented.

4. *The innovative techniques that are developed in the paper and their demonstrated advantages make it a valuable addition to the existing literature, providing a promising avenue for further research and practical applications in experimental design. In conclusion, considering the substantial contribution of this paper to the field of designs of experiments and its potential benefits to readers and practitioners, I strongly recommend the acceptance of this paper for publication.*

We thank the referee for the recognition of the contribution of our paper and for the suggestion to accept the paper.

**Response to reviewer 2.**

*Reviewer #2: Manuscript is well written and accepted in present form and accepted for publication only reference are outdated. Kindly add updated references. Also mentioned how we validate the new developed tool as previously RSM updated through p and R values.*

We thank the referee for the recognition of the contribution of our paper and for the suggestion to accept the paper as is.
As suggested, the reference have been updated to be current.
